# Unplanned Emergency and Urgent Care Visits After Outpatient Orthopaedic Surgery

**DOI:** 10.5435/JAAOSGlobal-D-21-00209

**Published:** 2021-09-20

**Authors:** Benjamin R. Williams, Lauren C. Smith, Arthur J. Only, Harsh R. Parikh, Marc F. Swiontkowski, Brian P. Cunningham

**Affiliations:** From the Department of Orthopaedic Surgery (Dr. Williams, Dr. Smith, Parikh, Dr. Swiontkowski), University of Minnesota, Minneapolis; the Department of Orthopaedic Surgery (Dr. Only, Dr. Cunningham), Methodist Hospital, St. Louis Park, MN; Department of Orthopaedic Surgery (Parikh), TRIA Orthopaedic Center, Bloomington, MN; and the Department of Orthopaedic Surgery (Dr. Only, Dr. Swiontkowski, Dr. Cunningham), Regions Hospital, St. Paul, MN.

## Abstract

**Introduction:**

This study sought to determine (1) incident risk, (2) chief report, (3) risk factors, and (4) total cost of unplanned healthcare visits to an emergency and/or urgent care (ED/UC) facility within 30 days of an outpatient orthopaedic procedure.

**Methods:**

This was a retrospective database review of 5,550 outpatient surgical encounters from a large metropolitan healthcare system between 2012 and 2016. Statistical analysis consisted of measuring the ED/UC incident risk, respective to the procedures and anatomical region. Patient-specific risk factors were evaluated through multigroup comparative statistics.

**Results:**

Of the 5,550 study patients, 297 (5.4%) presented to an ED/UC within 30 days of their index procedure, with 23 (0.4%) needing to be readmitted. Native English speakers, patients older than 45 years, and nonsmokers had significant reduced relative risk of unplanned ED or UC visit within 30 days of index procedure (*P* < 0.01). In addition, hand tendon repair/graft had the greatest risk incidence for ED/UC visit (11.0%). Unplanned ED/UC reimbursements totaled $146,357.34, averaging $575.65 per visit.

**Discussion:**

This study provides an evaluation of outpatient orthopaedic procedures and their relationship to ED/UC visits. Specifically, this study identifies patient-related and procedural-related attributes that associate with an increased risk for unplanned healthcare utilization.

As health care shifts toward a more value-based approach and certain orthopaedic procedures transition to the outpatient setting, information about postoperative healthcare resource utilization is critical to understanding the effectiveness of ambulatory surgery.^1,2,3,4^ Operative setting requires in-depth clinical decision making and assessment of potential risk to ensure safe administration of care. The Centers for Medicare & Medicaid Services recently estimated the national cost of readmissions, within 30 days after discharge, at $17 billion.^5,6^ Unplanned healthcare resource utilization has been extensively studied as a target for cost containment, but the focus has predominantly been on readmissions and/or inpatient orthopaedic surgeries.^3,4,7,8^ In addition, little is known about the unanticipated costs associated with outpatient surgery,^9,10^ especially for those who are seen in the acute healthcare setting, but not readmitted.^11,12^

The transition of procedures from the inpatient hospital setting to the outpatient ambulatory setting has required the meticulous assessment of risk versus value.^13^ Not all procedures, because of complexity, risk, and perioperative care requirements, are amenable to an outpatient setting. The extensive resources readily available in the inpatient setting necessitate that certain procedures continue to be done exclusively in the hospital. Continued evaluation of procedures that have been deemed reasonable to do in the ambulatory surgery is required to further assess if there are unanticipated concerns and cost. Multiple studies have sought to compare the frequency of unanticipated postoperative emergency department (ED) or urgent care (UC) visits between procedures done in the inpatient and outpatient settings.^14-16^ There exists a scarcity of literature evaluating risk factors prompting unplanned ED and UC visits after outpatient orthopaedic surgery and the accompany cost.

Identifying risk factors predictive of unplanned care utilization after outpatient surgery, as well as the cost of these visits, is essential as healthcare reimbursement continues to trend toward value-based payment models. Kelly et al^17^ conducted a study evaluating the most common complications and complaints leading to visits to the emergency department (ED) or urgent care visits (UC). Identification and evaluation of avoidable complications or diagnoses have been reported in the literature.^18^ The purpose of this study was to further examine unplanned healthcare utilization within 30 days after outpatient orthopaedic surgery and the additional associated cost of care. The primary outcome was (1) the incident risk of a visit to an ED or UC facility within 30 days of the index procedure. Secondary outcomes included (2) chief reports at ED/UC presentation, (3) evaluation of patient and procedural risk factors, and the (4) costs of unplanned healthcare visits estimated by the reimbursement. Our hypothesis was that outpatient orthopaedic trauma procedures would have the highest risks for unplanned healthcare utilization when compared with outpatient procedures.

## Methods

After Institutional Review Board approval, all patients treated within a large metropolitan healthcare system between 2012 and 2016 were retrospectively reviewed for an outpatient orthopaedic procedure. This healthcare system provides both insurance and care delivery to its patients. Patients included were 18 to 85 years, discharged on the same day as their procedure, and insured by the healthcare system. Unexpected admissions, before discharge from a planned outpatient procedure, were excluded. This was validated against the healthcare systems insurance database. Total joint arthroplasty (TJA) procedures were excluded because these procedures were not consistently being done in the outpatient setting of this healthcare system until 2016. Consequently, there were not an adequate number of procedures done for a meaningful analysis. Furthermore, in this healthcare system, most outpatient spinal procedures are done by neurosurgeons and not orthopaedic specialists and therefore were excluded as well.

Each procedure was processed as a unique episode of care and identified in the database through current procedural terminology (CPT) codes.^19^ The study cohort was first divided into procedural classifications as arthrodesis, arthroscopy, distal extremity, infection, and orthopaedic trauma. The arthroscopy grouping included all arthroscopic procedures involving the knee (anterior cruciate ligament [ACL] and meniscus), shoulder, (rotator cuff repair and labral repair), and elbow or hip. All hand and wrist and foot and ankle procedures that required an open surgical approach were classified under the distal extremity grouping. The trauma grouping included upper and lower extremity fracture care treated between day 1 to 3 weeks from injury. The study cohort was also divided into seven anatomical surgical regions: arm, foot and ankle, hand and wrist, hip, knee, leg, and shoulder and elbow. The healthcare system's electronic medical record (EMR) was queried to collect demographic characteristics including age, sex, body mass index, native language, the need for an interpreter, marital or living status, and smoking history (Table 1).

**Table 1:** Population Characteristics for Study Sample Between 2012 and 2016 (N = 5,492)

	n (% of N)	Mean + SD (95% CI)	
Same-day surgery procedural classification	Arthrodesis 157 (2.9%) Arthroscopy 1,882 (37.1%) Distal extremity 2,214 (40.3%) Infection 28 (0.5%) Trauma 1211 (22.1%)		
Same-day surgery procedural anatomical site	Arm 24 (0.4%) Foot and ankle 2,102 (37.9%) Hand and wrist 1,432 (25.8%) Hip 45 (0.8%) Knee 1,102 (20.1%) Leg 34 (0.6%) Shoulder and elbow 753 (13.7%)		
Sex	Male 2,410 (43.9%) Female 3,082 (56.1%)		
Age	45.8 + 16.1 (45.4, 46.2)		
BMI	29.1 + 6.6 (29.0, 29.3)		
Smoking history	Never 3,119 (56.8%) Former 1,457 (26.5%) Current 916 (16.7%)		
Living status	Single/divorced/separated 2,457 (44.7%) Married/partner/family 3,035 (55.3%)		
Language	English 5,272 (96.0%) Non-English 220 (4.0%)		
Interpreter needed	No interpreter 5,406 (98.4%) Interpreter needed 86 (1.6%)		
ED or UC visit within 30 days	No ED/UC visit 5059 (92.1%) ED/UC visit 433 (7.9%)		
ED/UC visit chief report	Medication-related concerns Swelling Wound check Pain Pain caused by reinjury Cast complications Adverse drug reaction (ADR) Urinary complications Others^a^ Unrelated visit		57 (13.2%) 43 (9.9%) 43 (9.9%) 39 (9.0%) 36 (8.3%) 36 (8.3%) 25 (5.8%) 12 (2.8%) 6 (1.4%) 136 (31.4%)

ED = emergency department, UC = urgent care

a Other ED/UC visits included case visits that were below five each. These cases included fever and shortness of breath.

A summary of study sample (N = 5492) characteristics. Proportions within parentheses are representative of only responses, excluding missing responses.

All ED and UC visits within 30 days from the date of surgery were defined as unplanned healthcare utilization. Unplanned visits to the surgeon's or primary care provider's office were not included in the analysis. These visits were recorded in the EMR as normal clinical visits, providing no method to identify them as unplanned or planned. Visits were analyzed using claims data and cross-referenced to match the patient's EMR for the original index procedure. Each patient's unplanned visit episode to an ED or UC was independently reviewed to assess the chief report and whether it was related to the index surgery. Finally, claims data were used to capture the reimbursement data associated only with the ED or UC visit.

**Figure 1:**
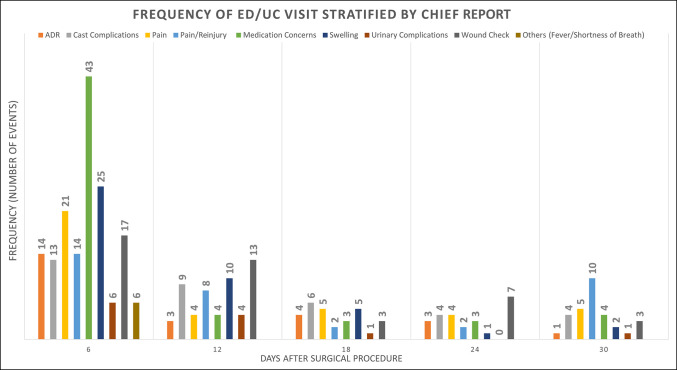
Bar chart showing the longitudinal 30-day postoperative rate of ED and UC visits, stratified by the chief report, measured in six-day intervals. ED = emergency department, UC = urgent care.

Descriptive statistics were used to report the incident risk and chief reports for unplanned visits after outpatient surgery (Table 1). To determine patient-related risk characteristics, comparative statistics were used to assess differences between cohorts who visited an ED or UC and those who did not. This included a combination of chi-square tests, Student two-sample *t*-tests, and multigroup analysis of variance *F*-tests (Table 2). A risk incidence matrix was constructed to evaluate the incident risk rates for ED or UC visits, respective to both the procedural classification and the anatomical region of the procedure (Table 3). Further analysis involved the formation of 18 groups arranged by specific CPT procedures. CPT procedures with fewer than 30 listed cases, less than 0.5% of the study population, were pooled into an “other” parent group. Finally, the 30-day ED/UC visit incidence was evaluated longitudinally, in days, visualizing by the primary chief report in 3-day intervals (Figure 1).

**Table 2:** Population Characteristics for Sample Population Between 2012 and 2016 Stratified by ED/UC Visit (N = 5492).

	No ED/UC Visit (n = 5195; 94.6%)		ED/UC Visit (n = 297; 5.4%)		*P*
Same-day surgery classification	Arthrodesis Arthroscopy Distal extremity Infection Trauma	152 (2.9%) 1,790 (34.5%) 2,095 (40.3%) 21 (0.4%) 1,137 (21.9%)	Arthrodesis Arthroscopy Distal extremity Infection Trauma	5 (1.7%) 92 (31.0%) 119 (40.1%) 7 (2.4%) 74 (24.9%)	**<0.01** ^a^
Same-day procedure anatomical site	Arm Foot and ankle Hand and wrist Hip Knee Leg Shoulder and elbow	21 (0.4%) 1,983 (38.2%) 1,353 (26.0%) 44 (0.9%) 1,048 (20.2%) 31 (0.6%) 715 (13.8%)	Arm Foot and ankle Hand and wrist Hip Knee Leg Shoulder and elbow	3 (1.0%) 119 (40.1%) 79 (26.6%) 1 (0.3%) 54 (18.2%) 3 (1.0%) 38 (12.8%)	0.54^a^
Sex	Male Female	2292 (44.1%) 2903 (55.9%)	Male Female	118 (39.7%) 179 (60.3%)	0.14^a^
Age	45.9 + 16.1 (45.5, 46.4)		43.5 + 15.4 (41.7, 45.2)		**<0.01** ^2^
BMI	29.1 + 6.5 (28.9, 29.3)		29.8 + 6.9 (28.9, 30.8)		0.12^2^
Smoking history	Never Former Current	2970 (57.2%) 1375 (26.5%) 850 (16.4%)	Never Former Current	149 (50.3%) 82 (27.5%) 66 (22.2%)	**0.02** ^a^
Living status	Single/divorced/separated Married/partner/family	2306 (44.4%) 2889 (55.6%)	Single/divorced/separated Married/partner/family	151 (50.8%) 146 (49.2%)	**0.03** ^a^
Language	English Non-English	5074 (97.7%) 121 (2.3%)	English Non-English	198 (66.7%) 99 (33.3%)	**<0.01** ^a^
Interpreter needed	No interpreter Interpreter	5117 (98.5%) 78 (1.5%)	No interpreter Interpreter	283 (95.1%) 14 (4.9%)	**<0.01** ^a^

ED = emergency department, UC = urgent care

a Resulting *P* value for a chi-square test between procedural groups.

b Resulting *P* value of the Student two-sample *t*-test between ED/UC visit groups.

All values that are in bold within the tables had p values <0.05. This was done to signify the statistical significance.

A summary of study sample (N = 5,492) characteristics. The number of reported 30-day ED/UC visits is related to only those that were determined to be related to the original index outpatient procedure. Summary statistics are provided in either count (proportion) or mean + SD (95% CI) format. The appropriate is used for each characteristic listed within the table. Proportions within parentheses are representative of only responses, excluding missing responses.

**Table 3:** Incidence Risk Matrix That Lists the Number of ED/UC Visits Within 30 Days of Their Respective Outpatient Orthopaedic Procedure (N = 5,492)

Anatomical location	Surgery Classification					
	Arthrodesis	Arthroscopy	Distal Extremity	Infection	Trauma	
Arm					3/24 (12.5%)	3/24 (12.5%)
Foot and ankle	3/120 (2.5%)	7/85 (8.2%)	68/1383 (4.9%)	4/16 (25.0%)	37/498 (7.4%)	119/2102 (5.7%)
Hand and wrist	2/37 (5.4%)	0/11 (0.0%)	51/831 (6.1%)	1/6 (16.7%)	25/547 (4.6%)	79/1432 (5.5%)
Hip		0/30 (0.0%)		0/1 (0.0%)	1/14 (7.1%)	1/45 (2.2%)
Knee		51/1053 (4.8%)		1/3 (33.3%)	2/46 (4.3%)	54/1102 (4.9%)
Leg				0/1 (0.0%)	3/33 (9.1%)	3/34 (8.8%)
Shoulder and elbow		34/703 (4.8%)		1/1 (100.0%)	3/49 (6.1%)	38/753 (5.0%)
	5/157 (3.2%)	92/1882 (4.9%)	119/2214 (5.4%)	7/28 (25.0%)	74/1211 (6.1%)	

ED = emergency department, UC = urgent care

A summary of the study population (N = 5,492) incidence for 30-day ED/UC return stratified by both the surgery classification and the anatomical location of the procedure. Each cell has the number of ED/UC visits, numerator, the total number of procedures done, denominator, and the resulting incident risk proportion.

Regression analysis consisted of multivariable logistic regression modeling the relative risk (RR) for 30-day return to the ED or UC. The model is stratified by their age, ordinal smoking status, primary English speaker, and their living status. Least absolute shrinkage selection operator regression was used to identify statistically relevant parameters for model adjustment.

All statistical analysis was conducted using SAS 9.4 (SAS Institute). The study data were compiled and maintained using Microsoft Excel within a secure hospital server (Microsoft Corporation). The level of statistical significance was set at *P* < 0.05.

## Results

A total of 5,407 patients with 5,492 unique outpatient surgical encounters were identified for the primary study cohort (Table 1). The study cohort was primarily female (*n* = 3,082, 56.1%), with a mean age of 45.8 years (95% confidence interval [CI], 45.4 to 46.2) and mean body mass index of 29.1 (95% CI, 29.0 to 29.3). The study population consisted of 1,457 former smokers (26.5%) with 916 current smokers (16.7%). Most of the study population, 3,035 (55.3%), identified as either married or living with a partner. A total of 220 participants (4.0%) identified as a nonnative English speaker with 86 (1.6%) requiring an interpreter (Table 1). Patients presenting to an ED/UC within 30 days were more likely to be younger (43.5 versus 45.9 years; *P* < 0.01), single/divorced/separated (50.8% versus 44.4%; *P* = 0.03), nonnative English speakers (33.3% versus 2.3%; *P* < 0.01), and active smokers (22.2% versus 16.4%; *P* = 0.02) (Table 2).

**Figure 2:**
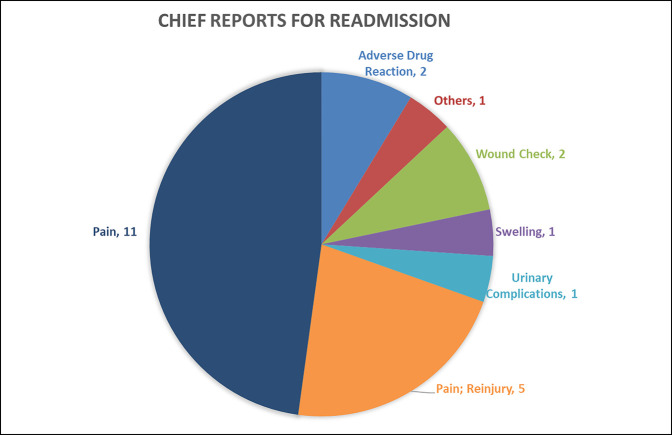
Pie chart showing the stratification of the chief report for ED and UC visits that required readmission. ED = emergency department, UC = urgent care.

**Figure 3:**
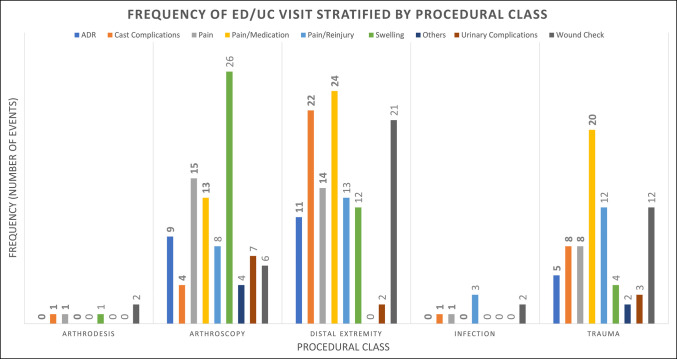
Bar chart showing the 30-day postoperative rate of ED and UC visits, stratified by the chief report and procedural classification. ED = emergency department, UC = urgent care.

A total of 433 patients (7.9%) presented to an ED/UC facility within 30 days of their index procedure; however, only 297 patients (5.4%) presented with a chief report relevant to their index procedure (Table 1). Most ED/UC visits (53.8%; 159 of 297) related to the index procedure occurred within the first 6 days after surgery (Figure 1). Of the 297 procedure-related ED/UC visits, 10.1% (30 of 297) occurred outside the primary health system. The three most common surgery-related chief reports were medication-related concerns (13.2%), swelling (9.9%), and wound concerns (9.9%) (Table 1). Overall, 0.44% patients were (23 of 5,492) admitted to the hospital or observation from the ED/UC (Figure 2) with a chief report related to their index surgery (6.7 days ± 8.1 [95% CI, 5.0 to 8.4 days]). Procedural classes of infection (25.0%; 7 of 28) and trauma (6.1%; 74 of 1,211) presented with the greatest incident risk of 30-day ED/UC return (Figure 3). In addition, anatomical regions of the arm (9.1%; 3 of 33) and leg (7.7%; 4 of 52) presented with the greatest incident risk of 30-day ED/UC return (Table 3). Being a native English speaker, age >45 years, and being a nonsmoker all contributed to a significant reduction in risk of an unplanned ED or UC visit within 30 days of index procedure (*P* < 0.01) (Table 4).

**Table 4:** RR Assessing for Relationships Between Demographic and Procedural Covariates and Return to the ED or UC Visit Within 30 Days of Index Procedure

Procedure Class	Beta (*β*) Estimate	RR	(95% CI)	Wald *X*^*2*^	*P*
Age (continuous)^a^	−0.01	0.99	(0.98, 1.00)	6.73	**<0.01**
Age (binary)^a^	−0.38	0.68	(0.55, 0.85)	11.51	**<0.01**
BMI	0.02	1.02	(1.00, 1.04)	2.48	0.12
Smoking (ordinal)	0.20	1.22	(1.02, 1.47)	4.50	**0.03**
Primary English speaking	−2.48	0.08	(0.07, 0.10)	592.07	**<0.01**
Living status^b^	−0.25	0.78	(0.59, 1.03)	3.04	0.08

BMI = body mass index, ED = emergency department, RR = relative risk, UC = urgent care

Logistic RR regression to assess the risk of a 30-day return to the ED/UC post-op with identified demographic and procedural covariates.

All values that are in bold within the tables had p values <0.05. This was done to signify the statistical significance.

a Age parameter was evaluated as both a continuous parameter and a binary parameter (reference age <45 years).

b Living status parameter was evaluated as a binary parameter; those living alone single/divorced/separated vs those living with another individual married/partner/family (reference).

RR regression found only infection-related procedures to have a statistically significant change in 30-day ED or UC return rate. After adjusting for age, smoking status, primary English speaker, and patient living status, infection-related procedures had a 3.53 times greater likelihood for returning to the ED or UC within 30 days of surgery (Table 5).

**Table 5:** RR Assessing for Relationships Between Procedural Subgroup or Anatomical Location and Emergency Department or Urgent Care Visit Within 30 Days of Index Procedure

Procedure Class	Beta (*β*) Estimate	Relative Risk	(95% CI)	Wald *X*^*2*^	*P*
Arthrodesis	−0.54	0.58	(0.24, 1.39)	1.49	0.22
Arthrodesis (adjusted)	−0.33	0.72	(0.23, 2.20)	0.33	0.56
Arthroscopy	−0.15	0.86	(0.68, 1.09)	1.50	0.22
Arthroscopy (adjusted)	−0.10	0.91	(0.65, 1.26)	0.32	0.57
Distal extremity	−0.01	0.99	(0.79, 1.24)	0.01	0.93
Distal extremity (adjusted)	−0.07	0.93	(0.68, 1.29)	0.18	0.67
Infection	1.55	4.71	(2.46, 9.03)	21.75	**<0.01**
Infection (adjusted)	1.21	3.53	(1.98, 5.72)	18.61	**<0.01**
Trauma	0.16	1.17	(0.91, 1.51)	1.50	0.22
Trauma (adjusted)	0.14	1.15	(0.80, 1.65)	0.55	0.46

ED = emergency department, RR = relative risk, UC = urgent care

Logistic relative risk regression to assess the relationship between various orthopaedic surgery classes and the risk of a 30-day return to the ED/UC post-op. Adjusting variables included continuous age, ordinal smoking status (never/former/current), primary English speaker, and living status.

The 297 unplanned ED/UC visits tallied to a total reimbursement of $146,357.34, averaging $575.65 per visit (95% CI, $494.84 to $656.46). The mean reimbursement (*P* = 0.39) was not significantly different between the procedural classes (Table 6).

**Table 6:** Mean Reimbursement for Visits to the ED/UC After 30 Days of an Outpatient SDS Orthopaedic Procedure Between 2012 and 2016 (N = 5,550).

Procedure	Count	Reimbursement	Standard Deviation	Range
Arthrodesis	5	$491.29	$400.67	$159.10, $936.26
Arthroscopy	92	$678.00	$667.94	$43.17, $3640.65
Distal extremity	119	$519.86	$670.90	$29.85, $3323.12
Infection	7	$904.35	$490.58	$346.43, $1268.25
Trauma	74	$514.65	$568.19	$14.36, $3111.75

ANOVA = analysis of variance, ED = emergency department, UC = urgent care, SDS = same-day surgery

Procedural cohort stratified reimbursements for 30-day ED or UC return after outpatient orthopaedic procedures within an SDS setting. The stratified means are identified as nonsignificant (*P* = 0.39), confirmed by a one-way ANOVA test.

## Discussion

This study demonstrates the importance of understanding the comprehensive episode of care. As healthcare reimbursement transitions toward bundled payments and value-based payment models, minimization of expensive and preventable healthcare utilization will be essential. This study sought to identify the rate and risk factors of unplanned healthcare visits when stratified by orthopaedic procedure types, identify common chief reports experienced within the initial 30-day postoperative period, and assess the overall cost burden for these healthcare visits.

Overall, 7.9% of patients presented to an ED or UC facility within 30 days after an outpatient orthopaedic surgery, with 5.4% of the visits being deemed relevant to the index procedure. Native English speakers, patients older than 45 years, and nonsmokers had a statistically significant reduced relative risk of unplanned ED or UC visit within 30 days of index procedure (*P* < 0.01). Contrary to our hypothesis, the trauma subgroup was not the highest incident risk of 30-day ED/UC return (6.1%; 74 of 1,211), superseded by infection (25.0%; 7 of 28). However, only 28 infection cases were evaluated in this investigation, compared with 1,211 trauma procedures, limiting the ability for statistical inference regarding incident risk. Among the remaining four procedural classifications, patients with trauma had the greatest risk of an ED/UC visit (Table 3).

Multiple studies have quantified the frequency of and identified risk factors contributing to unplanned ED/UC visits after outpatient orthopaedic surgeries.^11,18,20,21,22,23,24^ Although the recorded incidence of ED/UC visit is generally lower, variability exists within the literature.^11,18,20,21,22,23,24^ Sivasundaram et al.^20^ reported a slightly lower incidence of ED visit utilization (4.4%) within 30 days of outpatient surgery, compared with our study (5.4%), and 6.9% of patients presented to an unplanned ED within 7 days of surgery in the study of Navarro et al.. Contrary to our study, most unplanned ED/UC visits reported in the literature are accompanied by a chief report of postoperative pain.^18,20,21,23^ Only 9.0% of the patients presenting to an ED/UC visit in our study stated a pain-specific report. The most frequently reported reports prompting ED/UC visits in our study were medication-related concerns (13.2%). It is possible that the percentage of patients presenting to unplanned ED/UC visits secondary to a pain-related concern is underreported because of medication-related concerns not being stratified for pain medication-specific concerns.

This study demonstrates slight deviations with previous literature on unplanned healthcare utilization. The overall 5.4% ED/UC visit rate is greater than what has been previously reported in the literature, ranging between 3.0 and 4.4%, for both orthopaedic and general outpatient settings.^11,25,26,27,28^ This could be attributed to our inclusion of visits from outside institutions, captured through the insurance claims database. Cost analysis for unplanned orthopaedic care has focused primarily on hospital readmissions for hip and knee arthroplasty patients, with costs ranging from approximately $1,000 for a urinary tract infection to over $30,000 for a periprosthetic joint infection.^29,30,31,32^ Literature on cost for unplanned orthopaedic care in the ED or UC for outpatient procedures is sparse, although one arthroplasty study reported a total cost of $15,427^32^ for 36 unplanned ED visits that did not result in admission.^32^ This averages to a cost of $428.53/visit, less than our study's average reimbursement of $575.65/visit. This difference may be due to our study using reimbursement data to act as a surrogate of cost data.^33-35^

Using the results of this investigation, certain interventions can be postulated to reduce the use of unplanned visits. Phone calls, secure messaging through EMR, and earlier clinic visits customized around the patient's risk profile—smoking, marital status, or nonnative English speaker—could reduce ED/UC visits. The implementation of a phone consultation service was found to reduce ED utilization in TJA patients in Finland.^36^ Two recent American studies, one following surgical spine patients^37^ and the second TJA patients,^24^ found that increased postoperative utilization of outpatient orthopaedic clinic visits reduced the use of ED care.^38^ Given that half of the unplanned visits (53.8%) in our study transpired within the first 6 days from surgery,^11,32^ there may be an opportunity for early intervention for at-risk patients, possibly through an earlier follow-up appointment than the typical 10 to 14 days. Preoperative counseling to set clear expectations and clear discharge instructions are additional interventions that require minimal resources to implement and may limit visits to the ED or UC. The results of this study could be the stepping stone for the development of future interventions focusing on the outpatient orthopaedic surgical setting, maximizing cost savings and patient satisfaction.

To the best of our knowledge, this is the first study to use claims data within an insurance-owned healthcare system to reidentify all visits and to more comprehensively depict the true utilization of the ED and UC after outpatient orthopaedic surgeries. In addition, this study provides an evaluation of incident risk rates within the first 30 days of an outpatient procedure, risk rates by the procedural classification, anatomical regions, and the most common CPT codes. The utilization of insurer data also provides the ability to assess the relative costs of these visits. Finally, this study provided invaluable insight into patient-specific factors that can contribute to unplanned ED and/or UC visits after ambulatory surgery. As a result of our study findings, there have been increased institutional efforts into utilization of interpreter resources and more in-depth preoperative consultation before surgery. Consideration of patient home support and postoperative needs are thoroughly discussed before surgery and ancillary services, such as social work, are involved earlier in select cases. Ambulatory clinical support staff conduct postoperative follow-up calls to each patient within 48 hours of their procedure to assess how patients are recovering. In addition to these interventions, efforts are made to have each patient's initial postoperative visit within 7 to 10 days of their procedure. The application of our results is contributing to improved patient care and the utilization of healthcare resources.

This study has several limitations. First, the variability and limited documentation of provider–patient communication creates potential bias. It would be beneficial to have known whether providers or their staff contacted patients before their scheduled follow-up and whether patients contacted the nursing phone line before presenting to the ED or UC. This would also provide clarity whether patients used a provider's clinic instead of the ED or UC. Second, a nursing service line was introduced in December 2013. Before that date, 1,899 outpatient surgeries were done, and the risk for surgery-related unexpected healthcare visits was 5.2%. Of the remaining 3,704 outpatient surgeries done, the risk for unexpected healthcare visits was 5.4% (*P* = 0.87). However, there was an increase in unexpected healthcare visits, which was not statistically significant. In addition, there were not an adequate number of TJAs done in the study's time frame, nor a robust enough number of orthopaedic outpatient spinal surgeries for these procedures to be included. Therefore, the exclusion of spinal procedures and TJAs limits this study's generalizability. This study was unable to accommodate and analyze for unplanned visits to the provider's office or clinic. All visits to the office or clinic are scheduled in advance, whether it is a few hours or a few weeks, with no documentation of when the visit is scheduled. Therefore, no reliable method exists to discern whether an office visit was planned or unplanned. Future studies should be directed toward prospectively assessing other interventions that would be purposed at reducing unexpected healthcare utilization. In addition, the design of our study did not account for the potential confounding influence of comorbid conditions. It is possible that the incidence of ED/UC visits is secondary to patient-specific characteristics. We believe this study is an inaugural step in identifying factors that contribute to unplanned visits and utilization of healthcare resources after ambulatory surgery. Future investigation into identification of what specific patient comorbid conditions predispose patients to unplanned ED/UC visits after ambulatory surgery procedures is warranted. Finally, this study was conducted across a single healthcare system, and thus, the results may not be representative of other hospital settings and healthcare systems because of variance in patient populations, protocols, resources, and clinical staff. A future multicenter study would serve to evaluate variance in urgent care visits regionally after same-day surgical treatment.

We found that unexpected healthcare visits occur frequently. This study provides an overview of the patient-related and procedure-related risk factors that contribute to the utilization of these costly healthcare mediums. Using these results, future low-cost interventions could be formulated to target these areas and reduce overall healthcare cost. Future studies aimed at controlling the episode of care starting from the preoperative visit through the postoperative period will be important in managing costs, particularly with the growing utilization of outpatient orthopaedic surgery.
